# Understanding Patient and Clinician Perspectives in the HITH4Hips Trial: A Qualitative Study

**DOI:** 10.1111/ajag.70214

**Published:** 2026-08-02

**Authors:** Jananee Myooran, Justine Naylor, Marie K. March, Danielle Ní Chróinín, Thuy Anh Bui, Nicholas Bednorz, Shu‐Hsuan Chen, Bernadette Brady

**Affiliations:** ^1^ Geriatric Medicine Department Liverpool Hospital, South Western Sydney Local Health District Sydney New South Wales Australia; ^2^ School of Clinical Medicine, UNSW Medicine and Health UNSW Sydney New South Wales Australia; ^3^ Ingham Institute for Applied Medical Research Sydney New South Wales Australia; ^4^ Faculty of Medicine and Health, Sydney School of Health Sciences University of Sydney Sydney New South Wales Australia; ^5^ Physiotherapy Department Bankstown‐Lidcombe Hospital, South Western Sydney Local Health District Bankstown New South Wales Australia; ^6^ Physiotherapy Department Liverpool Hospital, South Western Sydney Local Health District Liverpool New South Wales Australia; ^7^ School of Health Sciences Western Sydney University Campbelltown New South Wales Australia

**Keywords:** healthcare acceptability, hip fractures, hospital to home transition, patient discharge, stay length

## Abstract

**Objectives:**

Early‐discharge models for hip fracture care are increasingly used to reduce hospital length of stay and healthcare burden, but stakeholder experiences remain underexplored. This study aimed to explore the experiences of patients, carers and healthcare providers (HCPs) involved in a multidisciplinary Hospital In The Home (HITH4Hips) program for low‐trauma hip fractures, and to identify factors influencing its implementation.

**Methods:**

Guided by the RE‐AIM QuEST (REach, Adoption, Implementation and Maintenance Qualitative Evaluation for Systematic Translation) framework, semi‐structured interviews were conducted with stakeholders involved in the HITH4Hips program at a metropolitan trauma hospital in Sydney, Australia. Interviews were transcribed verbatim and analysed using a rapid assessment approach with hybrid inductive–deductive coding. Themes were developed through interpretive analysis.

**Results:**

Nineteen interviews were completed, including 14 patients/carers (10 HITH4Hips participants; four usual care who declined HITH4Hips, mean age 75 years, 64% women, 86% from culturally and linguistically diverse background) and five HCPs. Four themes were developed: (1) Suitability for HITH: patients preferred home‐based recovery, whereas HCPs were more risk‐averse; (2) Perceptions of program effectiveness: patients prioritised emotional well‐being and functional progress, while HCPs emphasised measurable outcomes; (3) HCP mindset and orientation: role ambiguity and variable staff engagement limited program adoption; and (4) Realising program value: siloed communication and weak inpatient–community linkages hindered implementation.

**Conclusions:**

The HITH4Hips program was acceptable and has the potential to reduce healthcare system burden. Successful implementation will require tailored communication strategies and coordinated care pathways to align patient and provider priorities.

**Practice Impact**

Early‐discharge programs such as HITH4Hips are an acceptable and feasible way of reducing system burden. However, the success of implementation can be improved through shared vision and collaboration, a clear understanding of the program's purpose, as well as expanding the conceptualisation of effectiveness to include patient‐centred outcomes.

## 1 Introduction

Hip fractures are a leading cause of morbidity and mortality among older adults, with substantial consequences for both patients and healthcare systems [1]. In 2019, global hip fracture rates in adults aged 55 years or older were primarily caused by falls, with a prevalence of 1191 and an incidence of 681 per 100,000 population [2]. Incidence rises sharply with age due to declining bone fragility and higher fall risk, making hip fractures a significant public health concern [3]. In addition to clinical impact, hip fractures impose a considerable economic burden, driven by prolonged hospital stays, often reaching 14 days, and higher treatment costs [3, 4, 5]. In Australia, the average cost exceeds A$25,000 per case, approximately three times higher than for comparable patients without fractures, placing substantial pressure on healthcare resources and patient flow [4, 6, 7, 8].

To address these challenges, alternative care models, such as early supported discharge, for example, the Hospital In the Home (HITH) program, have been developed to safely reduce hospital length of stay while supporting recovery at home [9]. Evidence suggests that such models can improve patient outcomes, enhance satisfaction and reduce healthcare utilisation [10, 11, 12, 13]. National survey data indicate that HITH or Rehabilitation in the Home (RITH) program are offered in approximately two‐thirds of responding Australian hospitals, although none are specific to hip fracture patients [14]. Hospitals providing these programs reported shorter acute length of stay compared with those without (9.4 vs. 11.3 days, *p* = 0.04). Program uptake was lower in hospitals with higher proportions of culturally and linguistically diverse (CALD) patients or those requiring interpreter services [14]. Despite strong evidence supporting the effectiveness of these models, access and uptake remain variable [10]. There is limited understanding of the experiences of patients, carers and healthcare providers (HCP), particularly in CALD populations, and of the factors influencing the implementation of these programs at new sites.

The HITH4Hips feasibility trial was designed to examine the implementation of a new multidisciplinary early‐discharge model at a metropolitan site [15]. This was a single‐arm feasibility study in which eligible patients with low‐trauma hip fractures (LTHF) were offered participation in a HITH program, whereas those who were ineligible or declined received usual postoperative care. The HITH4Hips program was physiotherapist‐led and delivered coordinated medical, nursing and occupational therapy care in the community following early discharge.

This qualitative study, embedded within the HITH4Hips feasibility trial, aimed to explore the experiences of patients, carers and HCPs, and to examine contextual factors influencing the acceptability and implementation of the HITH4Hips program within a metropolitan CALD population [16]. By capturing patient perspectives, understanding HCP engagement and identifying barriers and enablers, the study sought to generate actionable insights to improve patient‐centred care and enhance the effectiveness of such early supported discharge programs for hip fracture patients.

## 2 Methods

### 2.1 Design and Setting

We conducted a prospective, single‐arm, quasi‐experimental study to evaluate the feasibility of the HITH4Hips program at Liverpool and Fairfield Hospitals, guided by the RE‐AIM evaluation framework [17]. Prior to rollout, a multidisciplinary team comprising medical, allied health, nursing and consumer representatives developed the HITH4Hips program components. Participation in HITH4Hips was a clinical decision made between patients/carers and the treating team; those who declined received usual postoperative care. The program targeted adults aged 50 years or older with low‐trauma hip fractures, focusing on early discharge, home‐based rehabilitation and patient safety. Of 101 patients screened, 19 were enrolled; all feasibility thresholds were met, and the program was delivered safely with high patient and carer satisfaction.

The embedded qualitative study used semi‐structured interviews with patients, carers and HCPs to explore program experiences and contextual factors influencing implementation [18]. Adopting a pragmatic epistemology, we used semi‐structured interviews with patients, carers and HCPs to capture diverse perspectives on program feasibility and acceptability. To ensure representation of the CALD population in South Western Sydney, Australia, all interviews were conducted in English, with a NAATI (National Accreditation Authority for Translators and Interpreters) health language interpreter supporting participants from Vietnamese or Arabic backgrounds as needed. Ethics approval was obtained from the.

SWSLHD Human Research Ethics Committee (ETH00172), and all participants provided written informed consent prior to participation.

### 2.2 Participants and Sampling

This study included two participant groups: patients/carers and HCPs/organisational stakeholders. Carers acted as a support person or proxy for patients who could not participate, ensuring that their perspective was not lost and contributing to a diverse and inclusive sample. Patients were eligible if they were aged 50 years and over, had a low‐trauma hip fracture requiring surgical treatment at Liverpool or Fairfield hospitals, were able to provide informed consent, and spoke English, Vietnamese or Arabic. Carers had no age restriction and were eligible if they had lived experience caring for a loved one with a hip fracture. All patients who were eligible for the HITH4Hips program, whether or not they consented to participate in HITH4Hips, were invited to participate in the qualitative sub‐study. Patients and carers were excluded if either one was unable to consent or did not meet the eligibility criteria for the overarching feasibility trial. Healthcare practitioners and organisational stakeholders were eligible if they were directly involved in the delivery, coordination or management of the HITH program at Liverpool Hospital and provided informed consent. Participants were purposively sampled to ensure representation across all three groups, including a range of clinical roles, experience levels and CALD participants.

### 2.3 Data Collection

Sociodemographic data, of patient/carer participants, as well as details of their admission were collected as part of the large feasibility study. Two semi‐structured topic guides (see Appendix A) were developed, informed by previous research and structured using the RE‐AIM QuEST framework [16], then refined through consultation with the research team and consumer representatives. Separate guides were created for patients/carers and for HCPs/organisational stakeholders, with the patient/carer guide piloted with a consumer prior to data collection. All interviews were conducted by the lead author (J.M.), who had minimal prior knowledge of stakeholders. Interviews were conducted within 2 months of discharge, between August and December, ensuring that participants had an adequate experience on HITH to be able to discuss their experiences. Interviews were conducted either in person or by telephone, depending on participant preference and logistical feasibility.

All interviews were audio‐recorded with permission and transcribed verbatim by (S.‐H.C.); transcription accuracy was then independently reviewed by two authors (J.M. and S.‐H.C.). Due to time constraints, participant burden during early recovery and literacy considerations in our CALD population, transcripts were not returned for validation. Instead, member checking was conducted by the research team, and field notes taken during and after interviews were used to support interpretive analysis. Only English language content was transcribed for interviews conducted with the assistance of a health language interpreter.

### 2.4 Data Analysis

Interview transcripts were analysed using rapid assessment analysis (RAP) [19], a time‐efficient approach combining narrative description with systematic coding to generate actionable insights for research and implementation of HITH4Hips [20]. A RAP (see Appendices B and C) was developed by two authors after independently conducting open inductive coding of two transcripts per participant type. Sensitising concepts from open coding were reviewed, and a draft template was piloted on two interviews and refined through discussion with the broader research team. Successive transcripts were coded independently using a hybrid inductive–deductive approach, with deductive codes mapped to the RE‐AIM QuEST framework domains and inductive codes capturing developed themes [19]. Author pairs met regularly to review coding, consolidate a single template and construct a framework matrix to facilitate cross‐participant analysis, comparing experiences across HITH and non‐HITH patients, carers and staff. Interviews were analysed according to the stakeholder group for which they belonged (patient/carer, or HCP). For patient participants, collaborative decision making including senior qualitative researchers and expert clinicians identified that at 14 interviews, adequate information power was reached to address the study aims [21]. The NVivo software (QSR International) was used for data management, coding and synthesis. Reflexive memos, field notes and demographic information were integrated to preserve context and enhance trustworthiness. Themes were interpreted in relation to the acceptability of HITH4Hips, as well as barriers and enablers to its implementation within a CALD population, with findings contextualised against existing early‐discharge frameworks. Although member checking was attempted by inviting all patient participants to a Teams presentation of our findings, no participants elected to participate [22].

The lead author (J.M.) led the construction of a framework matrix to facilitate cross‐participant exploration of patterns, mapped to the RE‐AIM categories. Patterns were analysed across HITH, non‐HITH patients and staff groups. Guiding questions utilised during analysis compared experiences across HITH and non‐HITH. Conclusions were drawn collectively, and then findings contextualised within knowledge of existing early‐discharge frameworks.

## 3 Results

### 3.1 Patient Characteristics

Table 1 summarises the characteristics of patients and HCPs involved in the HITH4Hips qualitative study. Of the 101 participants in the larger feasibility trial, 22 consented to the pathway. Nineteen patients transferred onto the HITH4Hips program, with five lost to follow‐up due to personal time constraints. Reasons for declining included preference not to participate in research, fatigue post‐discharge or scheduling conflicts. Interviews were conducted within 2 months of discharge. A total of 14 patients and carers were interviewed—10 who enrolled in HITH4Hips and four who opted not to; 86% were from CALD backgrounds, and carers were primarily close family members, including partners, children and grandchildren. For HCPs, all eligible participants involved in HITH delivery were invited, including 27 medical staff, five nursing staff and six allied health staff. Five HCPs consented to interviews, of which three were allied health staff (two occupational therapists, one physiotherapist) and two were physicians (an orthopaedic surgeon and an orthogeriatrician). Interview length ranged from 10 to 30 min.

**TABLE 1:** Participant characteristics of patients involved in the HITH4Hips qualitative study.

	Participant (*n* = 14)
Age, mean (range)	75 (67–83)
Gender, *n* female (%)	9 (64)
HITH‐accepted patients, *n* (%)	10 (71)
Length of stay (LOS), median (IQR)	13.5 (10.25–17.75)
CALD background, *n* (%)	12 (86)
Charlson Comorbidity Index (CCI), median (IQR)	5 (3.25–5)
Clinical Frailty Scale (CFS), median (IQR)	4 (3–5)
ASA physical status, mean (SD)	2.75 (0.66)
Number of physiotherapy sessions prior to discharge, median (IQR)	9 (7–11)
Able to mobilise on day‐1 post op, proportion *n* (%)	7 (50)

Abbreviations: ASA, American Society of Anaesthesiologists; CALD, culturally and linguistically diverse; CCI, Charlson Comorbidity Index; CFS, Clinical Frailty Scale; HITH, hospital in the home; LOS, length of stay.

Several themes were constructed from the study, reflecting the experiences of patients, carers and HCPs.

### 3.2 Theme 1: Characteristics and Suitability for HITH4Hips

Participants enrolled in the HITH4Hips were frequently medically complex, as evidenced by high comorbidity, frailty and ASA physical status (Table 1). Most patient and carer participants expressed a clear preference for recovering at home, describing the hospital environment as stressful and anxiety‐inducing, whereas home was associated with comfort and a sense of normalcy. As Participant 12 (P12) reflected, ‘in hospital you're really very concerned and worried about everything, you know, worried about your health’, whereas being home ‘really helped me to relax and you know, feel comfortable at home’. Although some participants were initially anxious about going home earlier than anticipated, most ultimately preferred home recovery. P8 noted that their family member ‘wanted to go home earlier… he can't sleep at any time in the hospital’, whereas P4 described that her mother ‘didn't feel comfortable being discharged… she was still nervous after the surgery… she felt more safe in the hospital’.

Trust in HCPs and the health system was central to accepting an early‐discharge model. Some participants were initially hesitant, but a strong therapeutic relationship helped them view HITH4Hips as a safe alternative. P1 noted, ‘I do trust the service of the hospital’. Conversely, participants who had strong expectations regarding inpatient and outpatient care expressed hesitancy to participate in a novel program. Conversely, P6—who declined—stated: ‘Who is he? He's just a physio. He can't decide [when she should be discharged]’. Social support was a critical enabler of successful home discharge. For example, P5's sister‐in‐law, who occasionally assisted his wife (and carer), highlighted ‘He [the participant] needs [his wife] around him most of the time. He needs aid with a few things still around the house… he struggles’. Carer gender sometimes posed cultural barriers: P6's daughter explained that ‘being males, it was hard for them. They couldn't take her to the bathroom or shower’, underscoring that carer and patient gender could pose a barrier where personal care assistance is required.

Compared with patient and carer participants, most HCPs adopted a more risk‐averse orientation, often viewing patient complexity as limiting HITH4Hips eligibility. Cognitive impairment was particularly challenging. Some HCPs sought stricter definitions or eligibility thresholds, whereas therapists emphasised the variability and context dependence of cognitive capacity. HCP3 observed that cognitively impaired patients were difficult in terms of following instructions and learning new tasks. Healthcare Participant 1 acknowledged that.

If their cognition is slight cognitive impairment they could go home with HITH4Hips if they had the available support in place…but we've got to recognise that we're going to come up with other issues. I know using a different mobility aid may be more difficult for them.(HCP1)

This framing reflects study design constraints, as well as a likely knowledge gap within our healthcare system, on caring for cognitive impaired patients, highlighting the need for future adaptations to safely include this patient subset.

### 3.3 Theme 2: Conceptualisations of Program Effectiveness

For patient and carer participants, program effectiveness was defined less by measurable outcomes and more by subjective experiences that supported recovery. The home environment was repeatedly described as a catalyst for improvement, helping participants to regain independence and confidence. For example, P12's carer reflected that she could ‘get used to being in her own home environment but also getting the services and what not rather than being in the hospital’. Emotional well‐being was critical to recovery: the carer for P5 observed he *was* ‘not panicking, not so anxious’. The absence of hospital routines allowed therapy and daily activities to be done at the participant's own pace, and carers noted the added convenience of supporting recovery at home: ‘I think the programs are important because not only does it help the patient, but it helps the family’ (P14). Engagement in exercises was key to perceived progress: P12 shared, ‘Gradually as I went along, I was able to do my own breakfast… I started to do a little simple cooking’. Both HITH and non‐HITH patients desired more therapy, with P11 emphasising that HITH4Hips ‘should be a little bit longer’. Those with realistic expectations, or those that were given a clear idea of recovery post‐hip fracture by HCPs, perceived therapy more positively despite being limited by pain or weakness. Contrastingly, P9, who seemed to have a poorer understanding of a typical post‐hip fracture recovery and expectations, reported that improvement was *‘too slow’* and *‘not fast enough’* with some days being *‘very bad’*.

In contrast, HCPs conceptualised program effectiveness in terms of measurable outcomes, such as length of stay, functional scores and avoidance of complications. For example, HCP2 questioned the program's efficacy in the absence of clearly defined, multidisciplinary goals:

I think establishing [goals]… because I guess for half it had to be kind of nursing and allied together…so whether or not nursing needed to kind of figure out what their role was in all of this?(HCP2)

Healthcare practitioners tended to adopt a short‐term, safety‐oriented lens, sometimes doubting whether HITH4Hips was appropriate for a patient group perceived as medically complex:

I'm not really sure this is the… model of care for our types of patients – just because they're just medically not optimised at baseline. (HCP2)

As a result, HCPs often overlooked the extended trajectory of rehabilitation that patient participants considered essential for achieving full functional optimisation.

### 3.4 Theme 3: HCP Mindset and Orientation

Although they generally endorsed the program and would participate again, HCPs held mixed views about its value. Those HCPs not directly involved in program delivery often saw it as a pragmatic solution to bed pressures, whereas those managing day‐to‐day logistics questioned whether the program's benefits justified the additional responsibilities—including patient education, coordinating follow‐up, liaising with the multidisciplinary team, completing documentation and arranging referrals. For example, HCP3 identified that it was challenging to balance recruitment, consenting, home assessments and provision of personal falls alarm as part of the HITH4Hips program, and HCP2 reflected, ‘I think HITH4Hips helps coordinate early discharge, but if patients are already safe to go home, what is its purpose?’ Time constraints limited the scope of HCPs, and some tasks were perceived as frustrating.

There was a bit of contention around the personal alarms and whose role it was…I feel like that kind of was pushed and forced upon us.(HCP3)

Hospital‐based HCPs highlighted a default to traditional roles and rigid scopes of practice, finding it difficult to transition to the flexible approach more common in outpatient care. Lack of buy‐in for tasks, such as consenting participants created role ambiguity. As HCP4 explained:

There was some suggestion that the registrars weren't doing enough to recruit patients… but the registrar's perspective was that it was the physiotherapist's responsibility to identify if they were suitable.(HCP4)

Four HCPs indicated that uneven distribution of responsibilities made the physiotherapist a central coordinator, complicating discharge planning:

There were sometimes a bit more of a conflicting plan for discharge…patients were getting confused about what the plan was.(HCP2)

### 3.5 Theme 4: Lack of Shared Vision and Communication

Healthcare practitioners lacked a consistent vision for the HITH4Hips program, which limited clear communication with patient and carer participants and hindered efficient program delivery. Siloed professional boundaries meant that essential tasks, such as participant consent, education and recruitment, were not recognised as shared responsibilities. P7 was unaware they would receive physiotherapy at home: ‘I didn't know that this program was giving me a physio, I didn't know’, leading them to remain longer as an inpatient. P4 highlighted pressure participants sometimes felt: ‘My grandma herself didn't want to get discharged early… but they were kind of pushing for it to happen’.

Healthcare practitioners also noted the absence of clear communication pathways between inpatient and community teams, which complicated early‐discharge coordination. HCP5 identified that communication between community teams and registrars was ‘close to non‐existent’ and that community staff lacked access to hospital records. Healthcare practitioners indicated that shared understanding of the program would allow better communication with participants, more balanced distribution of responsibilities across the team and smoother coordination with community services. HCP2 identified the need for ‘a clear idea in terms of what is actually, for example, each role of the program… which patients would be eligible… and establishing each discipline's role’. Practical improvements—clearer orientation processes, a consistent provider for patient education and consent, and coordinated scheduling of home visits—were seen as key enablers for realising the full value of HITH4Hips.

## 4 Discussion

This qualitative study examined patient, carer and HCP perspectives on the implementation of the HITH4Hips program, and identified that the program was well received by patient and carer participants, who valued home as a familiar environment that facilitated recovery, enhancing autonomy, confidence and emotional well‐being, as well as enabling family involvement. These findings align with prior research demonstrating that home‐based rehabilitation fosters independence, emotional well‐being and patient satisfaction [23]. Although early supported discharge programs have been evaluated across various patient populations, studies specifically examining implementation outcomes for post‐hip fracture patients and their carers remain limited, highlighting the contribution of this study to a relatively sparse literature.

However, HCPs identified several barriers to the implementation of the HITH4Hips project, detracting from participant experience. Our study found that a lack of overarching understanding and belief in the benefit of the program among HCPs impacted program implementation, communication and coordination across hospital and community teams. This ultimately affected and limited the adoption and maintenance of the HITH4Hips program. Effective implementation requires a collective vision and collaborative approach to distribute responsibilities, clarify roles and optimise scheduling of visits and therapy [18]. Healthcare practitioners should perceive the benefits of early supported discharge to justify the impact on workflow [24]. Yujia et al. demonstrated the need for program HCPs to have a comprehensive understanding of transitional care, through learning resources and supportive training, to advocate for patients and ensure proper implementation [25]. Earlydischarge programs, when well supported, result in HCPs recognising its positive effects and improving adoption, patient empowerment and cross‐sectional collaboration [26]. Engaging patients and families in the consent and implementation process is likely to further support program sustainability and help identify gaps in care, preventing post‐discharge complications [27]. Evidence from an Australian study indicated that multidisciplinary collaboration to identify discharge barriers ensures effective, coordinated patient care [28]. Clear communication both within HCP teams, and between HCPs and patients/carers, prevents inconsistent messaging and reduces confusion during discharge planning [28].

There is also a need for an improved model of care when implementing an early discharge, home‐based program in a cohort of HCPs that work with inpatients and are therefore risk‐averse by nature [29]. When it came to opinion on patient eligibility for HITH4Hips, staff were hesitant about whether complex patient cohorts, such as those that are cognitively impaired, were appropriate for this program, potentially affecting reach and implementation. A successful, community‐based program should see patients as a product of their environment and implement multifaceted interventions that may cross ordinary disciplinary boundaries [30]. Inter‐ and intra‐team collaboration, as well as diversion from the usual hierarchical structure of a medical team, allows for more effective and expansive care delivery [29]. Therefore, fluidity in hospital and community‐based clinicians, with a focus on HCP education and familiarisation, would improve implementation of HITH4Hips. Healthcare practitioners should be trained and supported in taking a community‐based, generalist approach to participants. A positive relationship between acute and community teams is also essential to improved feasibility when implementing new programs [28, 31].

Regarding conceptualisations of program effectiveness, HCPs often emphasised measurable, short‐term outcomes, including length of stay, functional status and avoidance of complications. As a result of this, they often failed to recognise patient‐valued outcomes, such as autonomy and psychological well‐being as being relevant or necessary—potentially affecting program adoption and maintenance. This short‐term, safety‐oriented perspective occasionally conflicted with patients' longer‐term goals of regaining independence and function, and sometimes there was a lack of clarity regarding discharge planning. Due consideration needs to be given to patient‐reported outcomes that matter to patients who are recovering from hip fracture [32]. For example, early‐discharge programs helped with the psychological recovery post‐surgery and improved confidence [33, 34]. Incorporating patient‐focused outcomes, such as self‐efficacy or fear of falling could provide a better understanding of benefits patients consider important [35].

### 4.1 Strengths and Limitations

This study was unique in evaluating a potential solution for improving patient flow for patients with hip fracture through an acute hospital while capturing patient, carer and HCP perspectives on its implementation. Unlike studies focusing solely on quantifiable outcomes, this research identified factors that may improve adoption and highlighted the experiences of patients, carers and HCPs. A key strength was its evaluation within a diverse cohort, including CALD and multimorbid patients, representing one of the most complex hospital populations for early‐discharge programs.

Saturation was only reached for the patient subgroup—not for HCPs or carers. Instead, as a pragmatic study, it achieved a saturated understanding of the elements of HITH and implementation elements from other stakeholders. The use of interpreters for patients who spoke Vietnamese or Arabic added to the complexity of the interviewing process, especially in phone interviews, and may have impeded the participants' accounts of their perspectives. Furthermore, the interview transcription only allowed for English audio to be transcribed, potentially limiting the verbatim accuracy of the interview transcriptions. Finally, the findings from this study were restricted to stakeholders consenting to interviewing and largely inpatient and acute HCPs. Therefore, findings would not be reflective of healthcare professionals in other contexts or broadly representative of all patients and carers of people with hip fractures.

### 4.2 Future Directions

Although patients, caregivers and HCPs found early‐discharge programs, such as HITH4Hips acceptable, HCP perspectives highlight several factors that poorly affected implementation, including patient eligibility and characteristics, HCP mindset and collaborative approaches. Centralised coordination of participants, clear pathways for escalation and follow‐up and defined scopes of practice are essential to improve program delivery [27]. Healthcare practitioner education and training should foster a community‐based, generalist approach and enhance understanding of both patient‐ and system‐level benefits, despite the siloed nature of community services from hospital services in New South Wales, Australia. Incorporating patient‐reported outcomes, such as functional recovery, self‐efficacy and fear of falling, alongside traditional clinical metrics, ensures that outcomes valued by patients are captured [32, 33, 35]. Actively engaging patients and carers in consent, orientation and ongoing communication supports program adoption, sustainability and safe transition from hospital to home [30].

## 5 Conclusions

The HITH4Hips program was generally acceptable to patients and carers, who valued the opportunity for early discharge and home‐based rehabilitation, and reported positive experiences with the support provided. Patients preferred home as the recovery environment and expressed trust in the healthcare system, with patient complexity and CALD backgrounds not perceived as barriers to participation. Contrastingly, HCPs identified several key limitations to effective implementation, including differing perceptions of program benefits, a lack of shared understanding of the program's purpose and an acute hospital–oriented mindset that conflicted with the community‐based focus required. These findings underscored the importance of aligning staff understanding and expectations, strengthening communication and incorporating caregiver support to optimise the acceptability and implementation of early supported discharge models for hip fracture patients.

## Disclosure

Statement on the Use of Generative Artificial Intelligence (AI): The authors confirm that no generative artificial intelligence tools were used in the writing or preparation of this manuscript.

## Ethics Statement

Ethics approval for this study was obtained by the SWSLHD Human Research Ethics Committee (ETH00172).

## Conflicts of Interest

The authors declare no conflicts of interest.

## Data Availability

The data that support the findings of this study are available from the corresponding author upon reasonable request.

## Appendix A

### Semi‐Structured Interview Guide

**INTERVIEW GUIDE—HITH PATIENT/CARER**.

*Reword for patient/carer as appropriate*.

### Introduction

Introduce yourself, thank them, explain purpose of interview.

Curated questions:

1. Could you tell me a bit about why you or your carer agreed to participate in the hospital in the home program (HITH)? [REACH]
  Prompts:

- What were your thoughts about getting home earlier vs staying in hospital longer?
- What concerns, if any, did you have about going home as part of HITH?
- What benefits, if any, did you feel you would have from going home as part of HITH?

2 I want to talk now about your experience once you went home with HITH. Can you describe a typical day for you while on the HITH program? [IMPLEMENTATION]
  Prompts:

- Who did you see?
- What did you or your carer have to do as part of the program?
- How well‐tailored to your situation was the HITH program?

3 How would you describe your recovery while doing the HITH program? [EFFECTIVENESS]
  Prompts:

- *Have you gotten better/worse or the same?*
- *What helped your recovery?*
- *What hindered your recovery?*

4 Based on your experience with HITH, how would you change/improve the program for others in the future? [IMPLEMENTATION and MAINTENANCE and SUSTAINABILITY]
  Follow‐up:

- Would you recommend this program to a family/friend in a similar situation?
- What experience prompted this suggestion? How do you think this change might improve the program?

5 Programs like HITH cost money. What amount of money would you be willing to pay per week to receive care at home?
  Prompts:

- For aged care services, you have to pay some money out of pocket to receive services.
- For GP, you need to pay a portion out of pocket, in addition to another portion being covered by Medicare.
- Does this influence your thoughts of what copayment may be acceptable for HITH?

Follow‐up:

- What influences this decision?
- How much do you think the government should pay if they had to fund it?

6 What are your thoughts on the HITH4Hips introductory video?

Follow‐up:

- Did it help your understanding of the program? How?
- How can it be improved?

**INTERVIEW GUIDE—USUAL PATIENT/CARER**.

*Reword for patient/carer as appropriate*.

### Introduction

Introduce yourself, thank them, explain the purpose of the interview.

Curated questions:

1. Could you tell me a bit about why you or your carer decided not to participate in the hospital in the home program (HITH)?

Prompts:

- What are your thoughts about getting home earlier vs staying in hospital longer?
- What concerns, if any, did you have about going home as part of HITH?
- What benefits, if any, did you feel you would have from going home as part of HITH?

2 I want to talk now about your experience in hospital. Can you describe a typical day while you were in the hospital recovering from your hip fracture?

Prompts:

- Who did you meet first?
- Experience with ward allied health?
- Clarity on what to expect, information/education supplements

3 Follow‐up: how would you describe your recovery in hospital?
4 If you broke your hip again‐ and we hope you don't!—would you try HITH4Hips, or would you prefer to stay in hospital for your care again?

Prompt: Why?

**INTERVIEW GUIDE—STAFF**

### Introduction

Introduce yourself, thank them, explain purpose of interview.

Curated questions:

1. Describe your role as part of the HITH4Hips program.

Prompts:

- What did you do?
- Who did you interact with?

Pick and choose questions below depending on staff's role:

2 What were the barriers to enrolling patients into the HITH program? [REACH]

Prompts:

- What did patients/carers have questions about?
- What concerns were expressed by patients/carers?

Follow‐up:

- How can the enrolment process be altered to address these issues?

3 How well did the HITH4Hips program function? [EFFECTIVENESS and IMPLEMENTATION]

Follow‐up

- What made it run well?
- What factors impeded the program running well?
- What were some examples where the outcome wasn't as good? Why?

4 What was your experience, as a staff member, in the HITH4Hips program? [ADOPTION]
  Follow‐up
  - What aspects affected your role?
  - Would you want to continue your role in this program? Why?
  - Would you recommend this to your colleague? Why?
5 What components of the program helped to keep it going? [MAINTENANCE]

Follow‐up

- Were any changes made to the program?
- What, in your opinion, would be a barrier to keeping the program going?

6 Would you like to add anything else?

## Appendix B

### Rapid Analysis Template

**HITH4Hips Analytical Template 1‐Patient/Carer**.

**Table:**

Key Information
Participant ID
HITH involvement (Patient/carer OR staff)
HITH‐accepted/not accepted
Initials of person preparing template

Domain	Key points and exemplar quotes
Patient characteristics + reach For this domain explore the individual characteristics of patients recruited to [or declining] the HITH program and factors that influenced their decision to participate or not. Consider what the interview tells us about: What personal factors contributed to or underpinned someone's decision to participate (or decline). Personal attributes of the participant (e.g., their cognition, health beliefs, health literacy, locus of control, past experiences). What implication does this (these characteristics and factors) have for appropriateness of the HITH program. How do personal factors influence someone's decision to participate?	
Effectiveness This domain explores what the participant perceives constitutes an effective program or experience AND how they interpret their experience against this perception (i.e., did they perceive their experience of HITH to be effective). Consider: How do they define effectiveness (e.g., do they interpret effectiveness in terms of measurable physical or psychological gains). How do they perceive their outcome following the HITH program? What influenced the outcome they achieved (barriers or enablers).	
Implementation This domain relates to how this program was integrated into the existing model of care and what modifications may be necessary from the patient/carer perspective. Consider: What did patients/carer understand about the program and its core components before commencing—was the program implemented according to their expectations? (e.g., Was inpatient care mirrored if this was an expectation). What reflections do patients/carers have on the processes adopted to implement HITH? This could include processes related to transferring between settings, identification and management of complications if applicable. What modifications, if any, do patients/carers perceive need to be adopted to optimise the HITH program and its processes?	
Maintenance and feasibility This domain relates to patient/carer perspectives regarding the continuation of a HITH program. Would patients/carers recommend a HITH program to others? If a person were to be hospitalised again, would they consider a HITH service again? Does the patient/carer consider HITH is an appropriate use of resources? How does the patient/carer reflect on the cost value of the program and the thought of co‐contribution?	
Any other points you consider to be significant to note about the implementation of the HITH program and the patient/carer experience not captured above	

## Appendix C

Rapid Analysis Template

**HITH4Hips Analytical Template—Other Stakeholders**.

**Table:**

Key Information
Participant ID
HITH involvement (Patient, carer, staff)
HITH‐accepted/not accepted
Person preparing template

Domain	Key points and exemplar quotes
Patient characteristics + reach For this domain explore stakeholder's perspectives of the participants recruited to or declining HITH and their suitability. Reflect on as relevant to the participant level: What barriers to enrolment did they encounter/were they aware of? Were the participants recruited to HITH representative of the normal fracture population? For example: baseline health status, Cognition Family/carer support, Health literacy. What patient characteristics were best suited to program? What characteristics should exclude a patient from the program? Were patients on HITH appropriate from their perspective?	
Effectiveness This domain explores the stakeholder's interpretation of effectiveness and their perspective of whether this was achieved during this implementation. Reflect on: What constitutes effectiveness to this stakeholder (e.g., Physical or psychological improvement, cost‐savings and length of stay). In what way, if at all, did HITH deliver on their effectiveness criteria? For example, Did patients improve on the program and meet their expectations? Did the program meet the expectation of HCPs? What explains the variation in effectiveness outcomes (if any) observed between (a) HITH vs Non‐HITH patients AND (b) those within HITH?	
Healthcare Provider Adoption + Consent. This domain relates to the factors that may influence a healthcare providers or organisations adoption of HITH. Consider as appropriate: What influenced the organisations decision to implement HITH? (e.g., Previous experiences, evidence, grant opportunity, funding or other necessity). Were HCP roles clearly defined? What was required from the role? How did HCPs communicate and work together? Were HCP's referring patients to the program, as part of routine care? Were there any issues with HCP consent or patient enrolment?	
Implementation This domain relates to how this program was integrated into the existing model of care, the processes behind implementation and whether the program was implemented with fidelity/as intended. Consider: The participant's reflection on whether the program as implemented with fidelity/as intended (e.g., Did it mirror inpatient care if that was the intent). Were there modifications to program and why? Were there any barriers to implementations from a patient, healthcare provider/team or organisational/contextual perspective? & how do we address them? Were safeguards followed with fidelity (i.e., Complication identification and management).	
Maintenance and feasibility This domain relates to the participants’ perspective regarding continuation of the HITH program and its potential for sustainability. In what form are the components of HITH being sustained (if at all). Consider—who were the key staff that kept the program going? What modifications have been made to the program since completion of the study? Or what would the necessary modifications be? Is it feasible to continue this program with the current resources?	
Other key issues relating the implementation of the HITH program not captured above.	
